# Utilizing the density of inventory samples to define a hybrid lattice for species distribution models: DISTRIB‐II for 135 eastern U.S. trees

**DOI:** 10.1002/ece3.5445

**Published:** 2019-07-17

**Authors:** Matthew P. Peters, Louis R. Iverson, Anantha M. Prasad, Stephen N. Matthews

**Affiliations:** ^1^ USDA Forest Service, Northern Research Station Northern Institute of Applied Climate Science Delaware OH USA; ^2^ School of Environment and Natural Resources The Ohio State University Columbus OH USA

**Keywords:** Forest Inventory and Analysis, habitat suitability, importance value, species abundance, statistical prediction

## Abstract

1. Species distribution models (SDMs) provide useful information about potential presence or absence, and environmental conditions suitable for a species; and high‐resolution models across large extents are desirable. A primary feature of SDMs is the underlying spatial resolution, which can be chosen for many reasons, though we propose that a hybrid lattice, in which grid cell sizes vary with the density of forest inventory plots, provides benefits over uniform grids. We examine how the spatial grain size affected overall model performance for the Random Forest‐based SDM, DISTRIB, which was updated with recent forest inventories, climate, and soil data, and used a hybrid lattice derived from inventory densities.
2. Modeled habitat suitability was compared between a uniform grid of 10 × 10 and a hybrid lattice of 10 × 10 and 20 × 20 km grids to assess potential improvements. The resulting DISTRIB‐II models for 125 eastern U.S. tree species provide information on individual habitat suitability that can be mapped and statistically analyzed to understand current and potential changes.
3. Model performance metrics were comparable among the hybrid lattice and 10‐km grids; however, the hybrid lattice models generally had higher overall model reliability scores and were likely more representative of the inventory data.
4. Our efforts to update DISTRIB models with current information aims to produce a more representative depiction of recent conditions by accounting for the spatial density of forest inventory data and using the latest climate data. Additionally, we developed an approach that leverages a hybrid lattice to maximize the spatial information within the models and recommend that similar modeling efforts be used to evaluate the spatial density of response and predictor data and derive a modeling grid that best represents the environment.

## 1 INTRODUCTION

Modeling a species' potential niche and mapping habitat suitability (HS) are standard practices for environmental research examining aspects of an ecosystem that influence species distributions, especially those impacted by ongoing change (Guisan et al., 2013). Such efforts can assist in defining a species' habitat range, be combined with field sampling to verify model performance, or aid in conservation and resource planning. Whether process‐based (Wang et al., 2014; Yospin et al., 2014) or statistical (Iverson, Prasad, Matthews, & Peters, 2008; Prasad, Iverson, Matthews, & Peters, 2016; Warwell, Rehfeldt, & Crookston, 2010), habitat modeling requires, at a minimum, information about the species' occurrence (i.e., importance or presence/absence, often from in situ plot data) and spatially indexed environmental conditions. Knowing what environmental information to include can be challenging in that variables are (a) scale dependent since values can differ at locations of species presence compared to aggregated values provided to the model (Kadmon, Farber, & Danin, 2003) and (b) must be relevant to the scope of the model, whether a macro‐ versus site‐level analysis or predicting occurrence versus abundance.

Models that encompass regional extents (e.g., areas greater than ~10,000 km^2^) generally rely on remotely sensed data representing environmental conditions, and the spatial resolutions of these datasets have improved over the past few decades (Pettorelli et al., 2014). Additionally, techniques have been developed to downscale and relate climate data to local scales (Daly et al., 2008; Wang, Hamann, Spittlehouse, & Murdock, 2012) and HS models are being developed at these finer resolutions (Franklin et al., 2013; Gottschalk, Aue, Hotes, & Ekschmitt, 2011). While it seems advantageous to model HS at the finest resolution possible, over large extents the resultant models may not adequately match the spatial density of inventory data used for model training, which often only represents a fraction of the modeling extent. Additionally, issues related to model extrapolation (see Dormann, 2007; Peters, Herrick, Urban, Gardner, & Breshears, 2004; Rastetter, 2017) need consideration related to what is being modeled.

The availability and accuracy of in situ inventory data collected by field sampling can be the biggest limitation to spatial resolutions when modeling over large extents. Field inventories are costly to implement at high densities, and thus, inventories may not fully sample representative habitats for rare species (Guisan et al., 2006; Mao & Colwell, 2005). Regardless of these drawbacks, inventory data are generally well‐suited for ecological modeling of habitats, provided positional errors are minimal (e.g., via data aggregation or smoothing) and/or modeling approaches that can reduce the influence of such errors are employed (Guisan et al., 2007). Dealing with locations where inventory data are not available can be done by omitting these locations from the model altogether or excluding them from the training dataset to then predict a value at these locations. However, the area of omitted or predicted locations can be quite large depending on the extent and grain size of the model in relation to the density of inventory samples.

We propose that the spatial density of inventory plots be used to develop a hybrid lattice of grid cells (Stevens, 1997; Tsui & Brimicombe, 1997) for summarizing model predictor variables. The U.S. Department of Agriculture, Forest Service Forest Inventory and Analysis (FIA) dataset is a systematic random sample of forest conditions with one survey plot per ~2,428 ha (Bechtold & Patterson, 2005); however, due to the spatial distribution of forestland, densified sampling in some states from state‐funded sampling, and the randomness of plot locations, each cell within the modeling lattice will have a varying number of inventory plots. One solution is to use Thiessen or Voronoi polygons to provide spatial structure among inventory plots, where irregular‐shaped polygons containing a single data point at their centroid partition the landscape. Holland, Aegerter, Dytham, and Smith (2007) examined regular and irregular geometries for use in modeling movement across a landscape and concluded that irregular geometries reduced bias resulting from the spatial structure representing the landscape. Irregular geometries are ideal for nonparametric point‐pattern analyses (Boots, 1980; Vincent, Haworth, Griffiths, & Collins, 1976), since no assumptions about the statistical distribution of response and dependent variables are made. However, a drawback to Thiessen polygons is that in regions that are sparsely sampled, large polygons represent a single inventory plot which may be uncharacteristic of the total area (Wilkin, King, & Sheldon, 2009). Therefore, a gridded network may provide a better representation of landscape conditions irrespective of sampling densities since variance of environmental conditions within each grid is reduced compared to the entire extent.

In this paper, we propose that a hybrid lattice may incorporate benefits of both Thiessen polygons and uniform grid networks for HS modeling. We compare whether models parameterized with data summarized with nested grids of both 10 × 10 and 20 × 20 km cells perform better in terms of model accuracy and reliability than 10 × 10 km uniform grid models. Concurrently, the evaluation of the hybrid lattice provides an update to our HS model, DISTRIB (described below), in which DISTRIB‐II attempted to model 135 tree species of the eastern United States.

## 2 MATERIALS AND METHODS

### 2.1 Species distribution model parameterization

The HS model, DISTRIB, uses FIA (www.fia.fs.fed.us) data to derive individual tree species importance values (IV, i.e., weighted abundance; Curtis & McIntosh, 1951) which are correlated to environmental conditions using Random Forest (RF hereafter, Iverson et al., 2008; Prasad, Iverson, & Liaw, 2006). It used a grain size of 20 × 20 km to summarize 38 environmental variables and aggregate species IVs, generally among two or more inventory plots, within the eastern United States (Iverson et al., 2008). DISTRIB has been used to predict potential current and future HS for 134 tree species under various scenarios of climate change; outputs are available from the Climate Change Tree Atlas (www.fs.fed.us/nrs/atlas, Prasad, Iverson, Peters, & Matthews, 2014) as are various vulnerability assessments (Brandt et al., 2017; Swanston et al., 2011) and general summaries of potential impacts (Iverson et al., 2017; Matthews & Iverson, 2017; Prasad et al., 2016).

**Figure 1:**
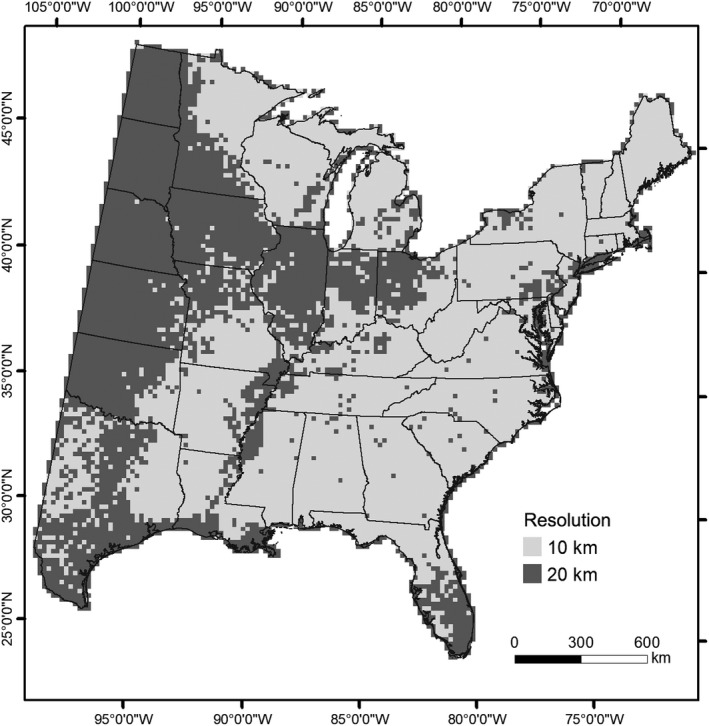
Extent of DISTRIB models and distribution of 10‐km and 20‐km grid cells within the hybrid lattice

We introduce DISTRIB‐II, which incorporates an overhaul of data sources, updates to the RF modeling technique, and the hybrid lattice approach of modeling. DISTRIB‐II takes advantage of the increased resolution of available environmental variables, and the newer and more comprehensive inventory data available through the FIA database. The FIA program collects and reports information about the nation's forest lands, and beginning in 1999, implemented annual inventories completed over a 5‐ to 7‐year cycle (O'Connell et al., 2017). However, due to insufficient funding, cycles for some states were extended. Sampling of forest conditions and individual trees ≥5.0 inches in DBH is performed on four 24‐foot radius subplots (O'Connell et al., 2017). For privacy, locations and information have been “fuzzied and swapped,” respectively (see Lister et al., 2005), and we use these records to calculate individual IV from the number of stems (e.g., relative density) and basal area (e.g., relative dominance). The resulting IVs range from 0 to 100 and were used as the response value for 135 eastern U.S. tree species (Appendix A1) and 45 environmental variables (Table 1) were aggregated from native resolutions to a 100 (10 × 10 km) and 400 km^2^ (20 × 20 km) lattice. Efforts to improve modeled HS by increasing the spatial resolution at which data are provided to RF have been conducted (Peters, Iverson, Prasad, & Matthews, 2013), and 84,204 annualized FIA records (Forest Inventory & Analysis Database, 2017) from the most recently completed cycle for 37 eastern states sampled during the period 2000–2016 were processed for DISTRIB‐II. Most states completed inventory cycles in 6 years initiated in 2005, 2007, or 2008; however, Louisiana, Texas, and West Virginia had longer cycles (11, 12, and 10 years, respectively). The underlying response grids used to develop HS models were refined to 10‐ and 20‐km grids (Figure 1), where each 20‐km cell was divided into four 10‐km cells. In addition to using a uniform grid (hereafter DISTRIB‐10), a hybrid lattice (hereafter DISTRIB‐hybrid), composed of 10‐ and 20‐km grids established by FIA plot density, was used to represent landscape conditions. An iterative algorithm determined whether sufficient FIA plots existed within each 20‐km grid to warrant increasing the resolution to 10 km. The 10‐km grids were accepted if ≥50% of the four 10‐km cells within a 20‐km cell contained two or more FIA plots; otherwise, the focal 20‐km cell was retained.

**Table 1:** Environmental data used to predict habitat suitability of eastern U.S. tree species. Data were either aggregated to 10‐ and 20‐km grids or derived from aggregated data

Category	Variable	Description	Native resolution
Climate^a^	[PANN] Annual precipitation	Mean 30‐year (1981–2010) monthly precipitation (mm)	800 m
	[PGrow] May‐September precipitation	Mean 30‐year (1981–2010) monthly precipitation for May–September (mm)	
	[TANN] Annual mean temperature	Mean 30‐year (1981–2010) monthly temperature (°C)	
	[TGrow] May‐September mean temperature	Mean 30‐year (1981–2010) monthly temperature for May–September (°C)	
	[TWINavg] Mean temperature of coldest month	Mean 30‐year (1981–2010) monthly temperature of coldest month (°C)	
	[TSUMavg] Mean temperature of warmest month	Mean 30‐year (1981–2010) monthly temperature of warmest month (°C)	
	[Aridity] Aridity Index	A conditional ratio of precipitation and Thornthwaite potential evapotranspiration (see Koch, Smith, & Coulston, 2013)	10 and 20 km
Elevation^b^	[ElvMIN] Minimum	Minimum value	90 m
	[ElvMEAN] Mean	Mean value	
	[ElvMAX] Maximum	Maximum value	
	[ElvMEDIAN] Median	Median value	
	[ElvMIN] Range	Range between minimum and maximum values	
	[ElvStdDev] Standard deviation	Amount of deviance among elevation	
	[ElvCV] Coefficient of variation	The CV of elevation	
Solar^c^	[DayLenCV] Day length coefficient of variation	The CV of 12‐monthly day lengths derived from the latitude of grid cells	10 and 20 km
Soil^d^	[AWC] Available water capacity (cm)	The quantity of water that the soil is capable of storing for use by plants	30 m
	[AWS] Available water supply (cm)	The total volume of water that should be available to plants when the soil, inclusive of rock fragments, is at field capacity	
	[BD3RDBAR] Bulk density (g/cm^3^)	The ovendry weight of the soil material <2 mm in size per unit volume of soil at water tension of 1/3 bar	
	[CACO3] Calcium carbonate	The percent of carbonates, by weight, in the fraction of the soil <2 mm in size	
	[CEC7] Cation‐exchange capacity	The total amount of extractable cations that can be held by the soil, expressed in terms of milliequivalents per 100 g of soil at neutrality (pH 7.0) or at some other stated pH	
	[DEP2WATTBL] Depth to water table (cm)	Depth to a saturated zone in the soil	
	[KSAT] Permeability (cm/hr)	Saturated hydraulic conductivity or the ease with which pores in a saturated soil transmit water	
	[KFACTRF] Erosion K factor	The susceptibility of a soil to sheet and rill erosion by water estimated by percentage of silt, sand, and organic matter and on soil structure and saturated hydraulic conductivity	
	[TFACTOR] Erosion T factor (tons/acre/year)	An estimate of the maximum average annual rate of soil erosion by wind and/or water that can occur without affecting crop productivity over a sustained period	
	[CLAY] Percent clay	Mineral soil particles that are <0.002 mm in diameter	
	[SAND] Percent sand	Mineral soil particles that are 0.05–2 mm in diameter	
	[SILT] Percent silt	Mineral soil particles that are 0.002–0.05 mm in diameter	
	[OM] Organic matter content (% by weight)	Plant and animal residue in soil material <2 mm in diameter at various stages of decomposition	
	[PH] pH	A measure of acidity or alkalinity	
	[SIEVE10] Percent passing sieve No. 10	Soil fraction passing a number 10 sieve (2.00 mm square opening)	
	[SIEVE200] Percent passing sieve No. 200	Soil fraction passing a number 200 sieve (0.074 mm square opening)	
	[SProd] Soil productivity^e^	Productivity Index derived from family‐level soil taxonomy information	
	Soil taxonomic order	The percentage of each of nine taxonomic orders: Alfisols, Aridisols, Entisols, Histosols, Inceptisols, Mollisols, Spodosols, Ultisols, and Vertisols	
	Soil texture	The percentage of texture class as defined by USDA standard terms: clayey, loamy, sandy, or other	

a PRISM Climate Group (2014).

b Farr et al. (2007).

c Forsythe, Rykiel, Stahl, Wu, and Schoolfield (1995).

d Soil Survey Staff (2016).

e Schaetzl, Krist, and Miller (2012).

In DISTRIB‐II, RF models were developed using the randomForest library (Liaw & Wiener, 2002) in R version 3.1.1 (R Development Core Team, 2014). Mean IV among FIA plots within each grid cell was modeled with 45 environmental variables consisting of climate, elevation, and soil properties (Table 1) for the hybrid and uniform grids. Only species occurring in at least 60 grid cells were considered for HS modeling. For each species, cells were excluded from the training data if (a) fewer than two FIA plots were present, (b) forest cover was <5% defined by the 2006 NLCD (Fry et al., 2011) (classes 41, 42, 43, and 90), or (c) the mean IV was >1.5 times the interquartile range of all cell IVs for the species so outlier values would not influence the models. We excluded cells from the training dataset with <5% forest cover (i.e., highly agricultural regions) containing two or more FIA plots because environmental drivers in those cells are likely to only marginally relate to forest species. We excluded IV outliers because they are unlikely to represent the broad 100‐ or 400‐km^2^ area, likely representing an artifact of recent forest or land use change. RF was parameterized to generate 1,001 regression trees, use eight randomly chosen predictor variables at each node (i.e., mtry), and grow each regression tree with a minimum of 10 observations. We set mtry to eight instead of the default, one‐third of the number of predictors (15 in this case), because predictor set redundancy resulted in better model performance statistics with fewer variables used at each node. Once the RF model was trained, predictions of IV were made to all cells regardless of size for the hybrid lattice, whether sufficient FIA plots were present, or percent forest cover was less than five percent.

### 2.2 Modeling species' importance

Each of the 1,001 regression trees built by RF provides information about the predicted IV, and the default is to report the mean prediction. However, the resampling of only eight of 45 variables at each node can result in spurious trees due to, for example, omission of an entire class (e.g., climate); while this does not influence overall prediction (Breiman, 2001), outliers can influence prediction distributions at a given cell (Roy & Larocque, 2012). Therefore, we compared the mean predicted value to the median for each cell; if the median = 0 and among all 1,001 predicted values the coefficient of variation (CV) ≥2.75, then 0 was used as the predicted IV rather than the mean, which was 0 < IV_mean_ <8 among all species. This “mean–median” combination is a modification to the approach suggested by Roy and Larocque (2012) which limits the influence on outlier predictions, yet retaining some marginally suitable habitat (e.g., mean prediction). In doing so, it gives more weight to half of the forest predicting a zero compared to a few trees predicting values >0 when the deviation of values is 2.75 times greater than the mean.

### 2.3 Evaluating model performance

We assessed statistical performance, or model reliability (ModRel), for each species' model among the DISTRIB‐hybrid and DISTRIB‐10 with five variables: (a) a pseudo‐*R* ^2^ obtained from the RF model (RF *R* ^2^); (b) a Fuzzy Kappa (FK) comparing the imputed RF map to the FIA‐derived map (Hagen‐Zanker, 2006, 2009); (c) a true skill statistic (TSS) of the imputed RF, after removing records with very high CV (e.g., mean–median combination); (d) the deviance of the CV (CVdev) among 30 regression trees via bagging (Iverson et al., 2008; Prasad et al., 2006); and (e) the stability of the top five variables (Top5) from 30 regression trees (Iverson et al., 2008). The five variables were normalized to a 0–1 scale and weighted as follows to arrive at a final ModRel score: 0.33*RF *R* ^2^ + 0.33*FK + 0.11*TSS + 0.11*CVdev + 0.11*Top5 which gives more weighting to RF *R* ^2^ and FK, a primary performance metric and a comparison of predicted to observed values, respectively. Then, ModRel scores were assigned to one of four classes: high (ModRel ≥ 0.7), medium (0.7 > ModRel > 0.54), low (0.55 > ModRel ≥ 0.14), and unreliable (ModRel < 0.14). Any species with negative RF *R* ^2^ were deemed unreliable and excluded from HS modeling.

Described in Iverson et al. (2008), FK is derived from a cell‐by‐cell comparison between the FIA IV and RF‐modeled IV (see Prasad et al., 2006), producing a 0–1 scale where one is a perfect match. FK is a better measure than percentage correct because the Kappa statistics account for uneven quantities of classes (Hagen‐Zanker, 2006, 2009), while the “fuzzy” part considers the proximity between classes (e.g., IV 1–3 vs. IV 4–6), which are a closer match than classes farther apart (e.g., IV 0 vs. IV 21–30).

The variation among 30 regression trees via bagging allowed an assessment of the consistency of the model outputs and provided two components of the ModRel scoring (Prasad et al., 2006). With a stable model, the deviance explained among 30 regression trees would vary little while an unstable model would yield trees explaining varying degrees of deviance. The CVdev variable (CV among 30 models) was calculated by (a) taking the weighted sums of the predictor deviance explained for each of the top five predictors; (b) calculating the CV (0–1) among the 30 bagging trees; and (c) subtracting this value from one to obtain a 0–1 score with one being most stable. Thus, it considers the amount and consistency of contribution of the top five predictors.

The Top5 variable uses the rank order of the top (up to five) predictors to compare the top RF variables among the 30 regression trees (via bagging; Iverson et al., 2008). We chose five variables arbitrarily to represent the primary drivers of the model, though for some species, fewer than five variables were needed to create suitable regression trees among the two grain sizes. The 0–1 scale was derived by summing the inverse ranks and dividing by the perfect match sum of 15 (assuming five points for first variable and one point for fifth variable entered into RF or bagging model). A score of one indicated that all five variables match the order exactly between RF and a bagging output, while a score of zero indicated no matches of top variables.

True skill statistic indicates how well the predicted values correspond to observed data (Allouche, Tsoar, & Kadmon, 2006); however, it can only be calculated for cells that contain observed IV and not for those in which imputation was used to predict an IV. TSS is only informative for a portion of cells, and we calculated TSS from IV by assuming that IV > 0 represents a predicted presence and IV = 0 represents an absence for the species.

In addition to calculating the performance statistics, confidence values (Wager, Hastie, & Efron, 2014) were calculated as a percentage of the 1,001 predicted values within one standard deviation of the mean or the median absolute deviation for records utilizing the mean–median combination, respectively. These confidence values can then be mapped to reveal spatial patterns of performance.

### 2.4 Species' range, detection, and abundance

For each of the 135 species modeled, information related to the spatial distribution of FIA data was used to classify the (a) distribution as narrow or wide, (b) density of FIA plots (commonness) as dense or sparse, and (c) FIA mean IV (i.e., abundance) as high or low. These classifications allow us to collectively evaluate the quality of the models as well as generalize some species characteristics. A species' distribution was considered narrow if the area of grid cells with FIA IV > 0 occupied <10% of the eastern United States; otherwise, it was assigned as wide. The density of FIA plots was considered dense if ≥40% of FIA plots among grid cells with IV > 0 for the species reported presence; otherwise, density was assigned to sparse. The abundance was considered high for average IV ≥ 6.0, the median of mean IV where the species occurs among all species, and low for values <6. These three categories were combined and coded with the first letter, where WDH indicates the species has a *wide* distribution, *dense* FIA plot ratio, and *high* IV. Codes for species that were withdrawn due to poor performance metrics were appended with an “X.”

### 2.5 Predictor variable importance

An evaluation of predictor importance was performed as described by Iverson et al. (2008) where a variable importance index (VarImpIndx) was derived from the average of three normalized (0–100) scores: (a) the sum of predictor importance scores from RF (percent increase) across all species (SumVarImp); (b) the sum of the reciprocal of ranked predictor importance across all species (SumRankRecip); and (c) the frequency of the top 10 predictors with the highest importance across all species (FreqTop10).

## 3 RESULTS

### 3.1 Representing species importance

**Figure 2:**
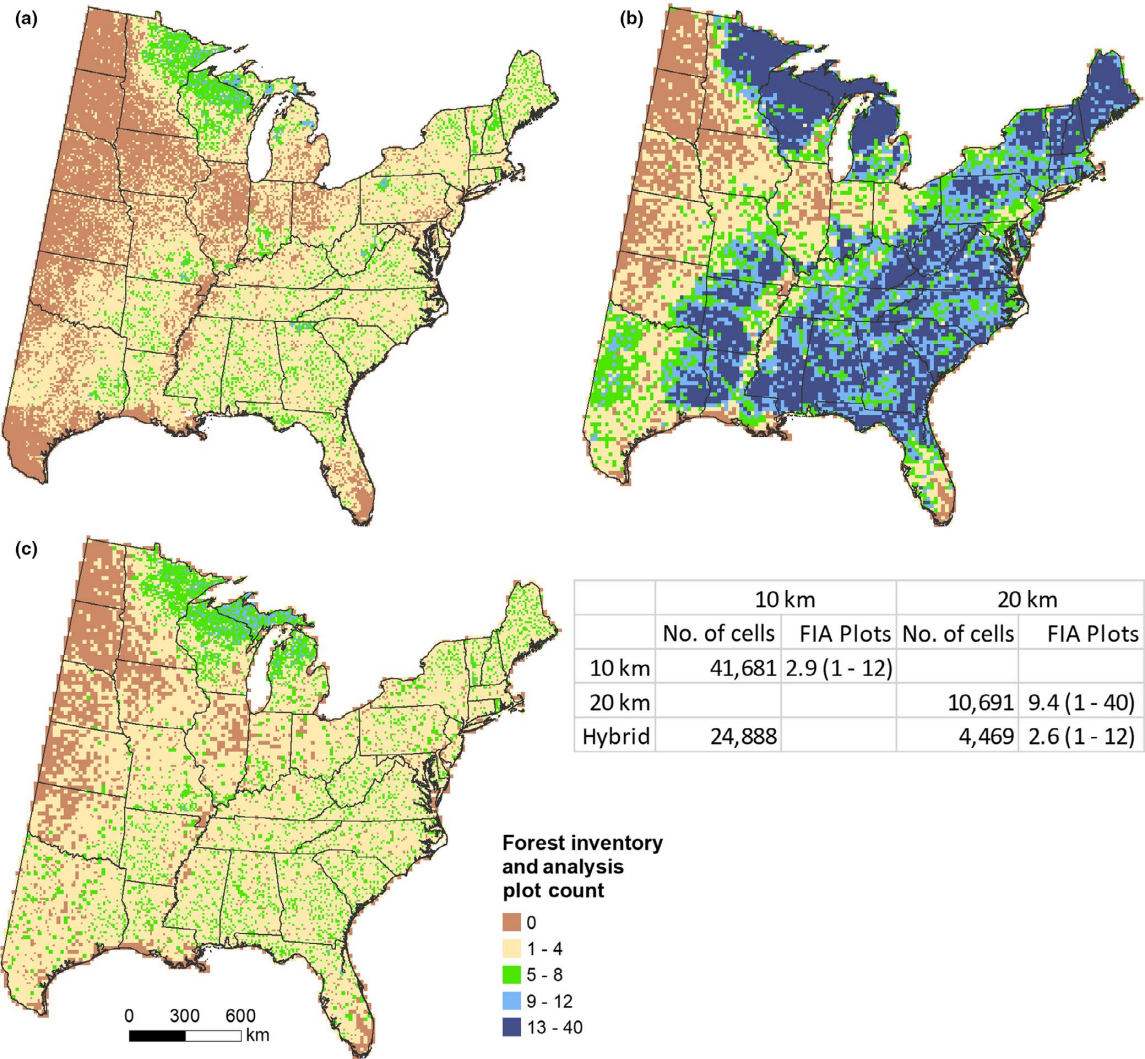
Count of Forest Inventory and Analysis (FIA) plots within (a) 10‐km grids, (b) 20‐km grids, and (c) hybrid lattice of 10‐ and 20‐km grids. The accompanying table includes the total cell count and mean (range) of FIA plots among cells

Across the eastern United States, uniform grids containing 41,681 and 10,691 cells had an average of 2.9 and 9.4 FIA plots at 10 and 20 km, respectively (Figure 2a,b). In contrast, the hybrid lattice contained 29,357 cells with an average of 3.2 FIA plots. Among the 20‐km grids, a maximum of 40 FIA plots occurred within some cells as a result intensified inventories. However, the hybrid lattice, of which 84.7% of the 29,357 cells are 10‐km grids, reduced the number of FIA plots to range from one to 12, with a mean plot count similar to the 10‐km uniform grid (Figure 2c). Thus, the hybrid lattice may provide a more representative sampling of the species' distribution across environmental conditions compared to a uniform 20‐km grid, which aggregates information from 2 to 40 plots for modeling.

### 3.2 Predicting species importance

**Figure 3:**
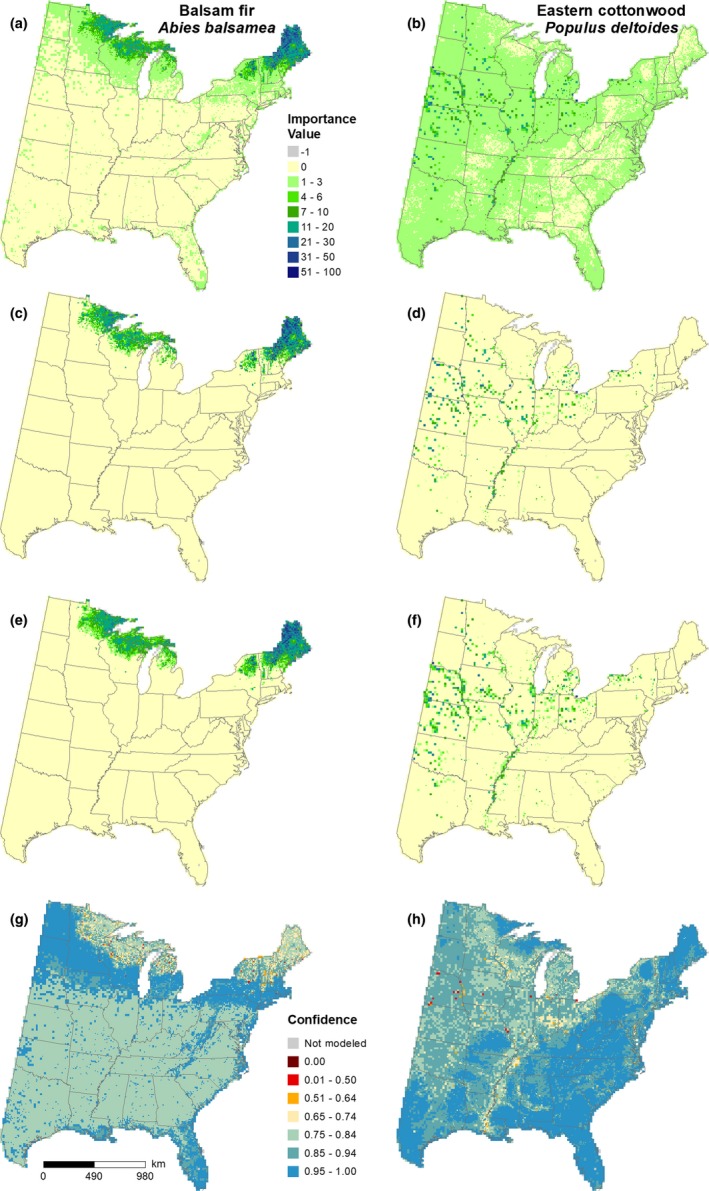
Modeled importance values for two species calculated by (a and b) the mean of 1,001 Random Forest (RF) predictions, (c and d) the median RF prediction, (e and f) a combination of mean and median RF predictions where the RF mean is used unless the median value is 0 and the coefficient of variation among predicted values is ≥2.75, then the median is accepted. (g and h) indicate the spatial confidence of RF predictions calculated as percentage of regression trees within ± 1 standard deviation of the mean or the median absolute deviation for the mean and the median prediction, respectively

**Figure 4:**
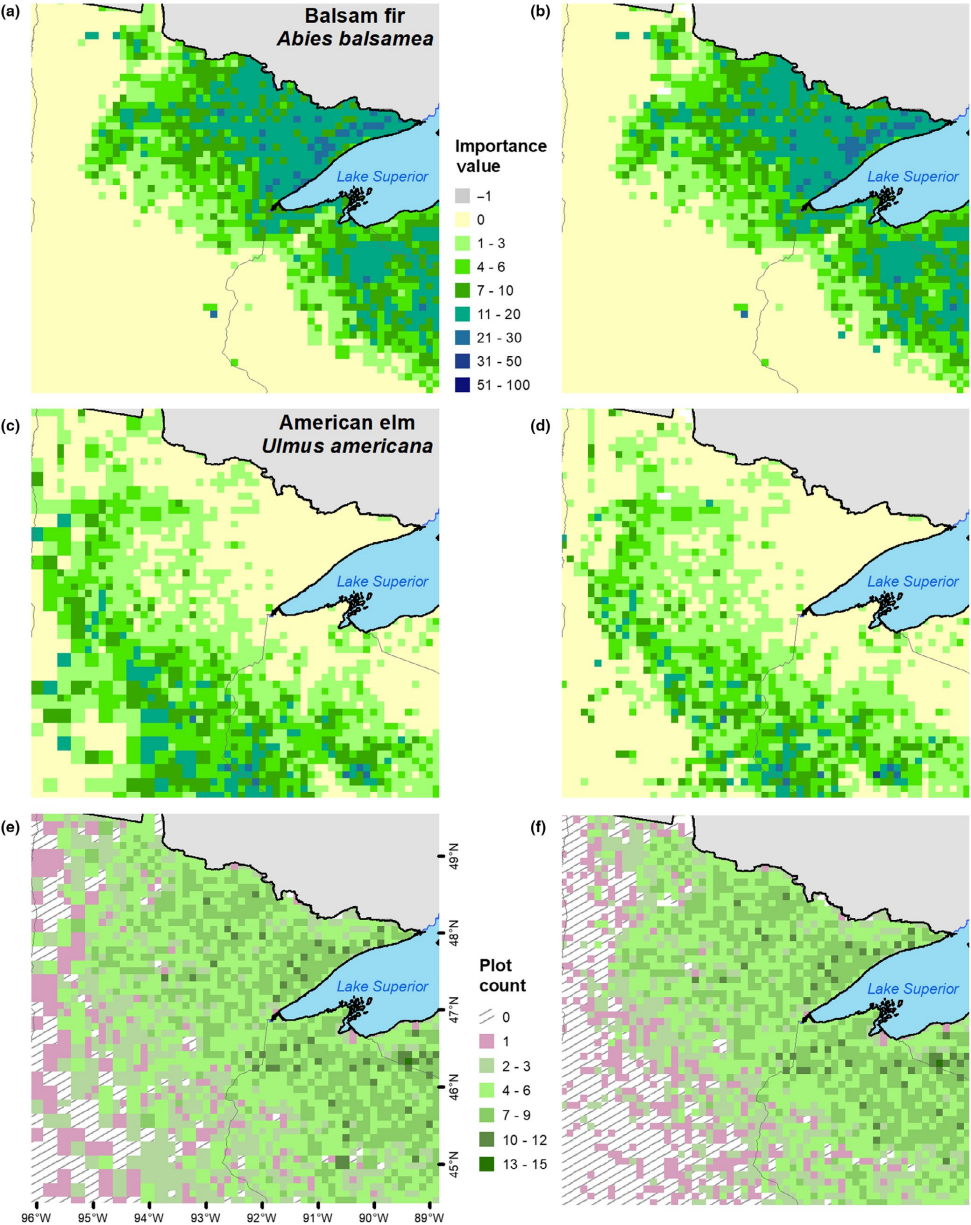
Comparison of predicted importance values between (a and c) the DISTRIB‐hybrid model and (b and d) DISTRIB‐10 model for a species corresponding to more 10‐km cells (*Abies balsamea*) and a species corresponding to more 20‐km cells (*Ulmus americana*). A comparison between the number of inventory plots per (e) the DISTRIB‐hybrid and (f) DISTRIB‐10 cells (see Appendix A4 for areal analysis)

For several species, the resulting DISTRIB‐hybrid models contained many mean predictions of relatively low IV in locations where a species would not be expected under current conditions (Figure 3a,b). When compared to the RF median prediction, these locations often had IV = 0 (Figure 3c,d); however, some of these locations might be suitable for a species though half of the 1,001 predicted values are zero. Combining the mean–median predictions ensures that some of these possibly suitable habitats remain (Figure 3e,f) in locations where the variance of predicted values varies little from the mean. Additionally, the confidence values provide a degree of certainty among the 1,001 predictions across the modeled spatial distribution (Figure 3g,h). Comparing predicted IVs between the DISTRIB‐hybrid and DISTRIB‐10 models for two species with differing range extents contrasts how the hybrid lattice informs the models (Figure 4). DISTRIB‐hybrid with 1,890 10‐km cells and 203 20‐km cells predicted an IV sum of 10,356 for *Abies balsamea*, while DISTRIB‐10 resulted in an IV sum of 10,426 (Figure 4a,b). Within the cells depicted in Figure 4, *Ulmus americana* has a predicted IV sum of 4,906 and 4,990 from the DISTRIB‐hybrid and DISTRIB‐10 models, respectively (Figure 4c,d). The modeling extent shown in Figure 4e,f had fewer cells containing <2 FIA plots for the DISTRIB‐hybrid model, an area of 58,500‐km^2^, compared to the 84,500‐km^2^ area within the DISTRIB‐10 model.

### 3.3 Evaluating model performance

**Figure 5:**
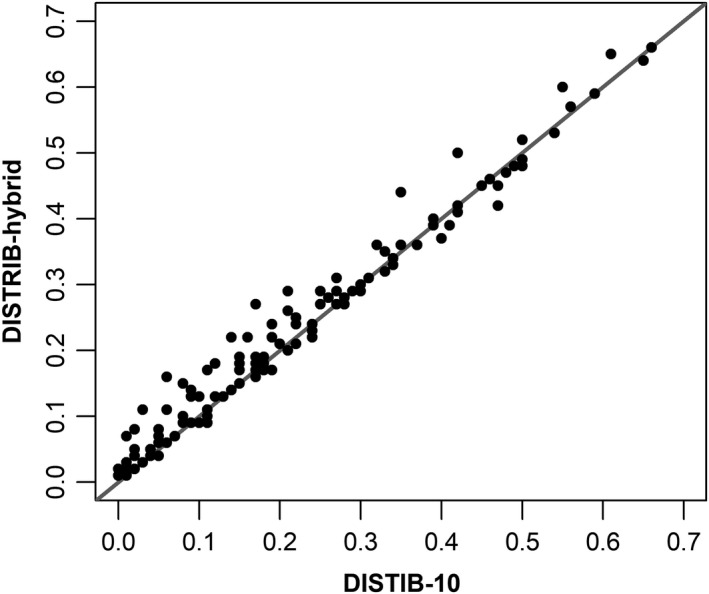
Comparison of Random Forest *R* ^2^ values for the DISTRIB‐10 and the DISTRIB‐hybrid models for 126 tree species with positive values. Points above the 1:1 line have a higher DISTRIB‐hybrid RF *R* ^2^ value compared to the DISTRIB‐10 models

The same modeling framework and underlying data were used to model HS for 135 species, and we examined how changes to the model's spatial resolution affected performance statistics (Table 2 and Appendix A1). Nine DISTRIB‐hybrid models were deemed unacceptable, having negative RF *R* ^2^ values, while one species had excessively low model reliability (ModRel < 0.14, Appendix A1); this left 125 modeled species. DISTRIB‐hybrid models resulted in higher RF *R* ^2^ than the DISTRIB‐10 models for 79 species, while the remaining 46 species were lower (Figure 5). TSS values were higher for 123 DISTRIB‐hybrid models, while FK values were lower for 124 species compared to the DISTRIB‐10 models (Appendix A1).

**Table 2:** Characterization of species' ranges and FIA records

CODE	Scale	Species count	Percent of species	RF *R* ^2^	FK	CV deviance	Top 5 variables	TSS	ModRel
NDH	10 km	18	13.3	0.494	0.923	0.879	0.475	0.845	0.740
	Hybrid			0.549	0.858	0.854	0.528	0.922	0.762
NDL	10 km	3	2.2	0.688	0.909	0.907	0.375	0.931	0.828
	Hybrid			0.691	0.936	0.905	0.759	0.906	0.871
NSH	10 km	24	17.8	0.329	0.875	0.684	0.320	0.797	0.623
	Hybrid			0.356	0.797	0.766	0.406	0.879	0.638
NSHX	10 km	1	0.7	0	0.828	0.496	0.288	0.587	0.409
	Hybrid			0	0.663	0.281	0.229	0.818	0.376
NSL	10 km	41	30.4	0.155	0.715	0.581	0.240	0.680	0.473
	Hybrid			0.171	0.689	0.619	0.293	0.718	0.473
NSLX	10 km	9	6.7	0.001	0.611	0.287	0.181	0.423	0.275
	Hybrid			0	0.49	0.306	0.161	0.603	0.263
WDH	10 km	19	14.1	0.56	0.851	0.925	0.394	0.845	0.74
	Hybrid			0.578	0.845	0.934	0.547	0.836	0.762
WDL	10 km	9	6.7	0.349	0.813	0.893	0.358	0.839	0.652
	Hybrid			0.350	0.839	0.891	0.542	0.795	0.667
WSH	10 km	4	3.0	0.325	0.843	0.796	0.358	0.800	0.629
	Hybrid			0.361	0.791	0.881	0.306	0.843	0.632
WSL	10 km	7	5.2	0.178	0.763	0.666	0.387	0.770	0.550
	Hybrid			0.201	0.775	0.679	0.440	0.760	0.550

Note

The range code field describes the distribution, density, importance, and model status. Distributions were narrow or wide, density is dense or sparse, importance is high or low, and model status was given an “X” if withdrawn (see text for assignment criteria). Mean scores (0–1) for RF *R* ^2^ obtained from Random Forest model, Fuzzy Kappa (FK) calculated on imputed mixed model predictions, coefficient of variation (CV) of deviance from 30 bagging models, top 5 variables from 30 bagging models, and true skill statistic (TSS) were used to derive model reliability (ModRel). See Appendix A1 for a list of species corresponding to the range codes.

The weighted metrics used for ModRel varied slightly among the two modeling grids. For DISTRIB‐hybrid models, 29, 47, 49, and 10 species were classified as having high, medium, low, and unacceptable ModRel, while the DISTRIB‐10 models resulted in 24, 43, 58, and 10 species, respectively (Appendix A1). Additionally, for all species, the DISTRIB‐hybrid models had higher mean confidence values, especially for species with high ModRel (Appendix A2).

### 3.4 Species' range characteristics

Among the 135 species modeled, 96 were considered to have narrow distributions within the eastern United States, 85 had sparse FIA densities, and 68 had low mean IVs (Table 2). Among the 10 species withdrawn resulting from narrow and uncommon distributions, one was from the NSH (i.e., narrow range, sparse density, high abundance) and nine were from NSL (narrow range, sparse density, low abundance) classes. Scaled RF *R* ^2^ and FK tended to be higher among species with dense FIA records rather than sparse records, especially for narrow distributions (Table 2). The NDL class (*N* = 3) had the highest ModRel (0.83–0.87) for each scale among all the distributional range classes, while the NSL class (*N* = 41) had the lowest ModRel (0.47). Overall, ModRel was generally higher (e.g., mean score of 0.76) for species with dense FIA records compared to species with sparse density of FIA plots (mean of 0.57). ModRel was also somewhat higher for species with high mean IV (0.69) versus low mean IV (0.64).

### 3.5 Predictor importance

The assessment of importance for 45 predictor variables placed TSUMavg, 30‐year mean temperature of the warmest month, as the most influential variable, followed by TWINavg, the 30‐year mean temperature of the coldest month (Appendix A3). The seven climate variables were in the top 50% of VarImpIndx scores, as well as day length CV and some soil properties (e.g., PH, SIEVE10, SProd, and KSAT). However, highly correlated variables, like some of the temperature variables, are often interchangeable within the RF models and likely provide the same information. The variables of elevation, soil properties, and types scored lower, having overall FreqTop10 and SumRankRecip scores under 40. Such lower values suggest that this type of information is often important for HS models of specific species or regions, but not so much across all species or the entire eastern United States.

## 4 DISCUSSION

### 4.1 The DISTRIB models

DISTRIB‐II, which will use the DISTRIB‐hybrid models, is a statistical model which predicts HS of individual tree species as IVs, based on inventory and environmental data. The spatial resolution of DISTRIB‐II has been increased where inventory densities are high by a hybrid lattice; however, each cell still represents a relatively large area (100–400 km^2^) for which the model output indicates a potential mean importance of a species. Assessing output from DISTRIB‐II at local sites is beyond the scope of the model as inventories within cells are aggregated to provide a representation of the species across the grid cell. Additionally, site‐specific conditions mediate establishment and competition among species (Clark, Gelfand, Woodall, & Zhu, 2014), which are only indirectly accounted for by inventory plots and are better assessed by modeling of colonization likelihoods (Prasad, Gardiner, Iverson, Matthews, & Peters, 2013; Prasad et al., 2016) and by local forest managers.

The DISTRIB‐II models are intended to provide macroscale information about the IVs for 125 tree species modeled within the eastern United States under current and future (see Iverson, Peters, Prasad, & Matthews, 2019) conditions. The models are highly dependent on FIA plot data, and the hybrid lattice approach is somewhat akin to the first DISTRIB models which predicted species IVs among counties using regression trees (Iverson & Prasad, 1998). While the underlying framework (e.g., statistical regression trees, climate, elevation, and soil data) of the DISTRIB‐II modeling approach is similar to previous versions, predicted values between DISTRIB versions will differ as a result of changes in source data and modifications to how environmental data were processed. Thus, each DISTRIB version represents a snapshot derived from current data and scientific knowledge, and one would expect each version to differ in the modeled spatial patterns and trends of abundance for each species. In our current efforts, differences arise from using a hybrid lattice approach, but also from (a) newer FIA records, (b) recent 30‐year climate normals, (c) a newer set of predictor variables, (d) removal of outlier training data, and (e) modifying predicted IVs with the mean–median combination. The FIA data from the most recently completed cycles were inventoried during the periods 2000–2016, and while many individual trees likely established several decades earlier, we chose the current 30‐year climate normal (i.e., 1981–2010) to account for recent changes and potential stressors that may be reflected by the inventories compared to earlier climate normals. Trees are long‐lived, and newly established individuals are likely responding to more recent climatic conditions and disturbances; using current conditions, we aim to capture these responses when exploring changes under future projections.

By refining the spatial resolution of DISTRIB‐II models and examining how cells size affected model performance, we have shown that for some metrics, the DISTRIB‐hybrid models had higher model performance as compared to the DISTRIB‐10 models. This behavior is likely due to added information in cells that would otherwise be excluded from DISTRIB‐10 training data because of too few FIA plots. However, by combining metrics that target specific aspects (e.g., presence/absence or fuzzy values) of the overall model's performance, the DISTRIB‐hybrid models for many species perform similarly or better than DISTRIB‐10. In the case of FK scores, however, the DISTRIB‐10 models were higher than the DISTRIB‐hybrid model, likely resulting from the increased number of cells and more closely predicted values between classes. Additionally, ModRel scores and the confidence values derived from RF predictions help to interpret potential responses from individual species by indicating overall confidence and where predictions agree or disagree.

### 4.2 Comparing the hybrid grid to other approaches

Thiessen polygons, though used in models for birds (Schlicht, Valcu, & Kempenaers, 2014; Wilkin, Perrins, & Sheldon, 2007) and amphibians (Holcombe, Stohlgren, & Jarnevich, 2007), like other irregular geometries used to model movement (Holland et al., 2007), have not been used generally to model vegetation patterns. This trend may be a result from the underlying vegetation inventory area having higher densities of sampling data or the plethora of gridded digital data available. Our use of a hybrid lattice borrows the spatial structure component of Thiessen polygons, but retains the uniformness of gridded data to stratify the landscape.

Approaches that use regional models (Ellenwood, Krist, & Romero, 2015) within a larger extent to predict local HS are useful when considering current ranges, but may not fully capture range‐wide conditions necessary to explore habitat changes arising from climate change. Therefore, we did not attempt to employ such techniques, but acknowledge that for current conditions, such approaches may improve model performance by reducing zero‐inflated datasets (Savage, Lawrence, & Squires, 2015). Other modeling efforts have produced predictions at spatial resolutions <500 m (Evans & Cushman, 2009; Rehfeldt, Worrall, Marchetti, & Crookston, 2015; Wilson, Lister, & Riemann, 2012) and as computational power increases and downscaled climate datasets become more widely available, this trend may become more prominent. However, while high‐resolution habitat models are desirable, the sampling density of inventory data remains a limitation in that sparsely sampled regions may not provide sufficient information for modeling.

A spatial grid, derived by the density of a focal object (e.g., FIA plots), may provide a more representative dataset for model training, and as such, we explored here the potential use of a hybrid lattice to model tree HS across the eastern United States, which has forest coverage ranging from nearly null in the “Corn Belt” to ~100% in the Appalachian Mountains. We found that the hybrid lattice capitalizes on the varying density of FIA plots to maximize the information content across the region; with ~85% of cells across the eastern United States having sufficient FIA plots for the 10‐km grid, we realize a fourfold increase in spatial resolution on tree species attributes over our earlier estimates with a 20‐km grid.

### 4.3 Transferability of modeling approach

Forest managers and decision makers often rely on modeled HS, primarily based on a uniform grid, which can have spatial resolutions too coarse for local needs. Although data aggregation techniques can readily process gridded and vector datasets independently, and methods exist to aggregate values between these two forms, results can include accuracy errors (Openshaw, 1983; Turner, O'Neill, Gardner, & Milne, 1989). The hybrid lattice approach presented here was applied to forest inventory data which was the main limitation to increasing the spatial resolution for our HS models. All environmental predictor datasets were available at native resolutions <1‐km grids. The process to derive the hybrid lattice can be useful for any information meeting specified criteria and applied iteratively to create multinested grids across the landscape.

Random Forest does not directly consider any spatial information (e.g., proximity or size), and our models were developed with tabular data, rather than gridded datasets. Other statistical techniques commonly used to model HS would have to accept tabular records or vector data, as raster data would have to be based on the smallest area within the hybrid lattice. A raster dataset would produce many duplicate values among the larger grids of a hybrid lattice and could influence the models similarly to zero‐inflated datasets by artificially increasing combinations of response and covariates. Thus, we believe the hybrid lattice approach presented here represents an improvement in DISTRIB‐II, and we advocate for its wider use in HS modeling.

## CONFLICT OF INTEREST

None declared.

## AUTHORS' CONTRIBUTIONS

MPP processed environmental data, modeled habitat suitability, and led the writing of the manuscript; LRI and SNM developed model reliability scores and contributed to the manuscript; AMP processed FIA records, provided guidance on Random Forest modeling, and contributed to the manuscript.

## Data Availability

Data presented in this paper will be made available at https://www.fs.usda.gov/rds/archive/, DISTRIB‐II: habitat suitability of eastern U.S. trees (https://doi.org/10.2737/RDS-2019-0029).

## APPENDIX A

**Table A1:** Model performance statistics for DISTRIB‐10 and DISTRIB‐hybrid models for 135 tree species. Model Reliability has been colored to indicate high (green), medium (yellow), low (pink), and unacceptable (purple). Species models having a negative RF R2 are colored red and Model Reliability is colored gray. Range class characterizes the overall distribution of the species range within the eastern United States

FIA Code	Scientific name	Range Class	DISTRIB‐10						DISTRIB‐hybrid					
			RF *R* ^2^	Fuzzy Kappa	TSS	CV dev	Top5	Model Reliability	RF *R* ^2^	Fuzzy Kappa	TSS	CV dev	Top5	Model Reliability
12	*Abies balsamea*	NDH	0.66	0.97	0.80	0.95	0.30	0.93	0.66	0.79	0.96	0.96	0.39	0.93
43	*Chamaecyparis thyoides*	NSH	0.11	0.89	0.58	0.66	0.16	0.50	0.11	0.54	0.86	0.67	0.22	0.47
61	*Juniperus ashei*	NDH	0.61	0.99	0.73	0.88	0.38	0.89	0.65	0.75	0.99	0.92	0.43	0.92
68	*Juniperus virginiana*	WDH	0.21	0.80	0.63	0.86	0.32	0.59	0.26	0.61	0.79	0.91	0.55	0.64
71	*Larix laricina*	NSH	0.45	0.90	0.70	0.84	0.40	0.78	0.45	0.68	0.9	0.89	0.61	0.80
94	*Picea glauca*	NSL	0.15	0.89	0.74	0.79	0.14	0.60	0.17	0.73	0.89	0.80	0.27	0.61
95	*Picea mariana*	NSH	0.50	0.92	0.72	0.89	0.31	0.80	0.49	0.70	0.92	0.85	0.48	0.80
97	*Picea rubens*	NDH	0.59	0.96	0.79	0.92	0.06	0.85	0.59	0.79	0.96	0.90	0.52	0.91
105	*Pinus banksiana*	NSH	0.34	0.94	0.70	0.80	0.16	0.68	0.34	0.66	0.94	0.82	0.20	0.66
107	*Pinus clausa*	NDH	0.33	0.93	0.64	0.80	0.29	0.68	0.35	0.62	0.93	0.72	0.43	0.68
110	*Pinus echinata*	WDH	0.49	0.89	0.70	0.90	0.30	0.78	0.48	0.71	0.88	0.86	0.55	0.81
111	*Pinus elliottii*	NDH	0.55	0.95	0.70	0.94	0.44	0.85	0.60	0.69	0.95	0.95	0.37	0.85
115	*Pinus glabra*	NSL	0.12	0.78	0.65	0.64	0.17	0.52	0.13	0.63	0.77	0.63	0.21	0.50
121	*Pinus palustris*	NSH	0.28	0.89	0.64	0.82	0.30	0.64	0.28	0.64	0.89	0.88	0.33	0.64
123	*Pinus pungens*	NSL	0.07	0.82	0.66	0.52	0.12	0.49	0.07	0.67	0.83	0.67	0.15	0.49
125	*Pinus resinosa*	NSH	0.27	0.92	0.64	0.89	0.48	0.68	0.27	0.62	0.91	0.91	0.33	0.63
126	*Pinus rigida*	NSH	0.50	0.86	0.68	0.73	0.19	0.74	0.52	0.67	0.86	0.83	0.37	0.77
128	*Pinus serotina*	NSH	0.34	0.84	0.64	0.71	0.38	0.67	0.34	0.63	0.84	0.79	0.43	0.67
129	*Pinus strobus*	WDH	0.37	0.89	0.68	0.93	0.13	0.68	0.36	0.66	0.88	0.93	0.36	0.70
131	*Pinus taeda*	WDH	0.65	0.92	0.74	0.97	0.29	0.88	0.64	0.74	0.91	0.96	0.37	0.89
132	*Pinus virginiana*	NDH	0.34	0.91	0.70	0.86	0.34	0.71	0.33	0.70	0.9	0.91	0.70	0.76
221	*Taxodium distichum*	NSH	0.22	0.86	0.65	0.82	0.13	0.58	0.25	0.63	0.87	0.78	0.23	0.59
222	*Taxodium ascendens*	NSH	0.30	0.92	0.68	0.78	0.33	0.68	0.30	0.65	0.92	0.75	0.31	0.65
241	*Thuja occidentalis*	WSH	0.39	0.94	0.69	0.90	0.36	0.74	0.39	0.69	0.93	0.92	0.40	0.74
261	*Tsuga canadensis*	NSH	0.37	0.91	0.69	0.91	0.03	0.67	0.36	0.69	0.9	0.94	0.38	0.72
311	*Acer barbatum*	NSL	0.15	0.61	0.58	0.58	0.09	0.46	0.15	0.60	0.62	0.72	0.19	0.47
313	*Acer negundo*	WSH	0.09	0.81	0.60	0.71	0.18	0.49	0.13	0.59	0.82	0.80	0.38	0.54
314	*Acer nigrum*	NSH	0.00	0.74	0.45	0.52	0.15	0.35	0.02	0.52	0.73	0.55	0.17	0.37
315	*Acer pensylvanicum*	NSL	0.28	0.77	0.63	0.87	0.18	0.59	0.27	0.63	0.77	0.87	0.45	0.63
316	*Acer rubrum*	WDH	0.47	0.71	0.68	0.95	0.32	0.73	0.42	0.67	0.64	0.95	0.39	0.70
317	*Acer saccharinum*	NSH	0.05	0.88	0.57	0.34	0.10	0.44	0.08	0.58	0.89	0.73	0.23	0.48
318	*Acer saccharum*	WDH	0.42	0.86	0.67	0.96	0.15	0.70	0.42	0.67	0.84	0.92	0.39	0.73
319	*Acer spicatum*	NSL	0.18	0.41	0.24	0.70	0.25	0.31	0.18	0.25	0.41	0.74	0.36	0.32
331	*Aesculus glabra*	NSL	0.02	0.80	0.56	0.57	0.23	0.44	0.05	0.58	0.77	0.34	0.14	0.40
332	*Aesculus flava*	NSL	0.08	0.79	0.61	0.54	0.10	0.46	0.09	0.64	0.8	0.61	0.10	0.47
356	*Amelanchier spp*.	NSL	0.09	0.45	0.35	0.53	0.13	0.29	0.09	0.34	0.45	0.44	0.15	0.26
367	*Asimina triloba*	NSL	0.01	0.54	0.30	0.27	0.07	0.22	0.01	0.31	0.52	0.27	0.10	0.20
371	*Betula alleghaniensis*	NDL	0.54	0.90	0.75	0.95	0.02	0.78	0.53	0.74	0.9	0.94	0.65	0.87
372	*Betula lenta*	NDH	0.39	0.94	0.75	0.92	0.20	0.75	0.40	0.74	0.93	0.90	0.27	0.75
373	*Betula nigra*	NSL	0.01	0.78	0.62	0.28	0.12	0.43	0.01	0.62	0.79	0.48	0.17	0.42
375	*Betula papyrifera*	WDH	0.46	0.94	0.77	0.93	0.30	0.81	0.46	0.77	0.93	0.93	0.19	0.78
379	*Betula populifolia*	NSL	0.11	0.69	0.64	0.65	0.18	0.49	0.10	0.64	0.7	0.66	0.19	0.48
381	*Sideroxylon lanuginosum ssp. lanuginosum*	NSL	0.02	0.73	0.47	0.39	0.12	0.36	0.04	0.52	0.71	0.33	0.17	0.36
391	*Carpinus caroliniana*	WSL	0.11	0.71	0.62	0.63	0.12	0.48	0.09	0.61	0.7	0.59	0.16	0.45
401	*Carya aquatica*	NSL	0.16	0.79	0.59	0.46	0.14	0.50	0.22	0.65	0.81	0.62	0.14	0.55
402	*Carya cordiformis*	WSL	0.05	0.78	0.59	0.46	0.30	0.47	0.06	0.61	0.78	0.54	0.27	0.46
403	*Carya glabra*	WDL	0.22	0.83	0.67	0.93	0.28	0.62	0.21	0.68	0.81	0.94	0.35	0.62
404	*Carya illinoinensis*	NSH	0.02	0.80	0.59	0.63	0.07	0.43	0.08	0.64	0.83	0.66	0.28	0.50
405	*Carya laciniosa*	NSL	0.02	0.76	0.47	0.17	0.17	0.36	0.02	0.46	0.76	0.19	0.10	0.31
407	*Carya ovata*	WSL	0.12	0.83	0.62	0.79	0.37	0.56	0.18	0.64	0.83	0.85	0.36	0.58
408	*Carya texana*	NDL	0.33	0.91	0.73	0.80	0.20	0.69	0.35	0.74	0.91	0.79	0.38	0.73
409	*Carya alba*	WDL	0.18	0.80	0.65	0.90	0.03	0.54	0.17	0.65	0.78	0.94	0.66	0.63
421	*Castanea dentata*	NSLX	0.01	0.37	0.09	−0.20	0.11	0.08	0.01	0.09	0.33	−0.28	0.15	0.02
452	*Catalpa speciosa*	NSHX	−0.01	0.82	0.47	0.48	0.18	0.38	−0.01	0.53	0.81	0.27	0.16	0.35
461	*Celtis laevigata*	NDH	0.17	0.81	0.62	0.75	0.12	0.53	0.27	0.63	0.8	0.80	0.47	0.64
462	*Celtis occidentalis*	WDH	0.08	0.82	0.58	0.70	0.35	0.51	0.15	0.61	0.84	0.85	0.31	0.55
471	*Cercis canadensis*	NSL	0.07	0.65	0.58	0.55	0.20	0.44	0.07	0.56	0.64	0.61	0.13	0.40
491	*Cornus florida*	WDL	0.19	0.75	0.66	0.69	0.11	0.55	0.17	0.64	0.74	0.63	0.53	0.58
521	*Diospyros virginiana*	NSL	0.01	0.51	0.54	0.46	0.06	0.34	0.02	0.58	0.53	0.57	0.11	0.36
531	*Fagus grandifolia*	WDH	0.41	0.83	0.68	0.91	0.05	0.68	0.39	0.67	0.81	0.92	0.45	0.73
541	*Fraxinus americana*	WDL	0.22	0.77	0.62	0.90	0.33	0.59	0.24	0.63	0.75	0.93	0.14	0.56
543	*Fraxinus nigra*	WSH	0.29	0.86	0.69	0.91	0.34	0.68	0.29	0.68	0.86	0.90	0.04	0.62
544	*Fraxinus pennsylvanica*	WSH	0.10	0.73	0.57	0.58	0.03	0.43	0.13	0.55	0.73	0.82	0.03	0.45
546	*Fraxinus quadrangulata*	NSL	0.06	0.74	0.56	0.63	0.17	0.44	0.06	0.61	0.76	0.58	0.18	0.45
551	*Gleditsia aquatica*	NSLX	−0.01	0.88	0.46	0.37	0.12	0.37	0.00	0.46	0.81	0.25	0.05	0.30
552	*Gleditsia triacanthos*	NSH	0.03	0.82	0.59	0.47	0.18	0.45	0.11	0.61	0.84	0.66	0.18	0.49
555	*Gordonia lasianthus*	NSH	0.17	0.86	0.64	0.76	0.40	0.60	0.18	0.66	0.86	0.74	0.33	0.58
571	*Gymnocladus dioicus*	NSLX	−0.01	0.83	0.47	0.38	0.05	0.35	−0.01	0.58	0.83	0.38	0.17	0.39
580	*Halesia spp*.	NSL	0.10	0.70	0.28	0.47	0.26	0.33	0.09	0.26	0.71	0.55	0.33	0.31
591	*Ilex opaca*	NSL	0.26	0.69	0.62	0.85	0.07	0.55	0.28	0.61	0.69	0.88	0.35	0.60
601	*Juglans cinerea*	NSLX	0.00	0.69	0.47	0.56	0.17	0.36	0.00	0.47	0.7	0.51	0.05	0.31
602	*Juglans nigra*	WDH	0.09	0.84	0.60	0.65	0.27	0.51	0.14	0.62	0.84	0.73	0.26	0.53
611	*Liquidambar styraciflua*	WDH	0.47	0.90	0.73	0.95	0.15	0.76	0.45	0.72	0.89	0.98	0.23	0.76
621	*Liriodendron tulipifera*	WDH	0.42	0.87	0.70	0.94	0.24	0.73	0.41	0.70	0.86	0.96	0.45	0.76
641	*Maclura pomifera*	NDH	0.06	0.89	0.60	0.70	0.27	0.50	0.16	0.61	0.9	0.71	0.31	0.55
651	*Magnolia acuminata*	NSL	0.17	0.76	0.56	0.77	0.03	0.48	0.16	0.54	0.77	0.81	0.29	0.50
652	*Magnolia grandiflora*	NSL	0.05	0.69	0.56	0.56	0.16	0.42	0.04	0.55	0.7	0.50	0.14	0.39
653	*Magnolia virginiana*	NSL	0.24	0.82	0.68	0.85	0.22	0.62	0.23	0.68	0.81	0.84	0.17	0.59
654	*Magnolia macrophylla*	NSL	0.18	0.47	0.20	0.51	0.13	0.27	0.18	0.16	0.46	0.50	0.08	0.22
655	*Magnolia fraseri*	NSL	0.14	0.79	0.63	0.52	0.29	0.54	0.14	0.61	0.79	0.56	0.28	0.50
682	*Morus rubra*	NSL	0.01	0.60	0.49	0.39	0.06	0.33	0.07	0.55	0.63	0.66	0.20	0.41
691	*Nyssa aquatica*	NSH	0.20	0.84	0.64	0.76	0.15	0.57	0.21	0.63	0.85	0.77	0.16	0.56
693	*Nyssa sylvatica*	WDL	0.24	0.82	0.70	0.90	0.20	0.63	0.22	0.70	0.8	0.93	0.53	0.66
694	*Nyssa biflora*	NDH	0.24	0.91	0.68	0.89	0.32	0.65	0.24	0.68	0.91	0.88	0.13	0.61
701	*Ostrya virginiana*	WSL	0.13	0.68	0.62	0.67	0.08	0.48	0.13	0.61	0.68	0.55	0.30	0.48
711	*Oxydendrum arboreum*	NDL	0.50	0.89	0.75	0.90	0.50	0.84	0.48	0.75	0.88	0.91	0.56	0.83
721	*Persea borbonia*	NSL	0.17	0.69	0.60	0.61	0.32	0.53	0.17	0.60	0.71	0.66	0.37	0.53
722	*Planera aquatica*	NSL	0.01	0.74	0.54	0.20	0.18	0.39	0.03	0.55	0.75	0.68	0.22	0.41
731	*Platanus occidentalis*	NSL	0.04	0.82	0.62	0.29	0.13	0.45	0.05	0.63	0.82	0.39	0.05	0.42
741	*Populus balsamifera*	NSH	0.32	0.87	0.69	0.78	0.32	0.69	0.36	0.68	0.87	0.76	0.25	0.68
742	*Populus deltoides*	NSH	0.01	0.87	0.61	−1.77	0.01	0.33	0.03	0.59	0.89	0.67	0.25	0.46
743	*Populus grandidentata*	NSL	0.18	0.86	0.66	0.85	0.30	0.60	0.18	0.66	0.85	0.83	0.34	0.59
746	*Populus tremuloides*	WDH	0.56	0.92	0.71	0.94	0.09	0.79	0.57	0.70	0.91	0.94	0.17	0.80
761	*Prunus pensylvanica*	NSL	0.02	0.47	0.40	0.61	0.06	0.28	0.02	0.40	0.48	0.64	0.28	0.31
762	*Prunus serotina*	WDL	0.25	0.68	0.61	0.85	0.64	0.64	0.27	0.60	0.64	0.85	0.29	0.57
763	*Prunus virginiana*	NSLX	−0.02	0.34	0.27	0.38	0.08	0.18	−0.02	0.33	0.35	0.50	0.11	0.20
766	*Prunus americana*	NSLX	−0.01	0.46	0.17	0.23	0.09	0.15	−0.01	0.23	0.46	0.06	0.03	0.12
802	*Quercus alba*	WDH	0.40	0.77	0.66	0.94	0.02	0.65	0.37	0.65	0.74	0.95	0.36	0.68
804	*Quercus bicolor*	NSL	0.00	0.84	0.56	0.56	0.12	0.42	0.01	0.58	0.84	0.48	0.16	0.41
806	*Quercus coccinea*	WDL	0.30	0.88	0.69	0.90	0.15	0.65	0.30	0.69	0.87	0.84	0.32	0.67
809	*Quercus ellipsoidalis*	NSH	0.31	0.91	0.70	0.83	0.13	0.66	0.31	0.69	0.91	0.80	0.24	0.66
812	*Quercus falcata*	WDL	0.21	0.86	0.69	0.89	0.07	0.59	0.20	0.70	0.84	0.87	0.44	0.64
813	*Quercus pagoda*	NSL	0.18	0.85	0.65	0.73	0.32	0.59	0.19	0.66	0.86	0.77	0.26	0.58
816	*Quercus ilicifolia*	NSLX	0.00	0.65	0.44	−0.14	0.21	0.31	−0.01	0.41	0.6	0.08	0.07	0.23
817	*Quercus imbricaria*	NDH	0.11	0.89	0.62	0.76	0.08	0.51	0.17	0.70	0.88	0.44	0.35	0.58
819	*Quercus laevis*	NSH	0.22	0.82	0.66	0.74	0.17	0.58	0.21	0.64	0.85	0.77	0.15	0.57
820	*Quercus laurifolia*	NDH	0.27	0.85	0.67	0.90	0.37	0.66	0.29	0.67	0.85	0.91	0.36	0.66
822	*Quercus lyrata*	NSL	0.15	0.85	0.64	0.68	0.14	0.54	0.19	0.65	0.86	0.68	0.26	0.57
823	*Quercus macrocarpa*	NDH	0.19	0.88	0.63	0.85	0.45	0.62	0.24	0.61	0.89	0.85	0.18	0.58
824	*Quercus marilandica*	NSL	0.21	0.77	0.64	0.80	0.19	0.56	0.29	0.68	0.77	0.84	0.19	0.62
825	*Quercus michauxii*	NSL	0.03	0.69	0.60	0.59	0.08	0.42	0.03	0.60	0.7	0.54	0.04	0.39
826	*Quercus muehlenbergii*	NSL	0.17	0.85	0.65	0.80	0.09	0.54	0.19	0.64	0.84	0.81	0.23	0.57
827	*Quercus nigra*	WDH	0.33	0.90	0.72	0.96	0.38	0.73	0.32	0.72	0.89	0.96	0.39	0.72
828	*Quercus texana*	NSH	0.15	0.83	0.57	0.59	0.10	0.49	0.18	0.61	0.84	0.55	0.34	0.54
830	*Quercus palustris*	NSH	0.06	0.92	0.63	0.70	0.16	0.50	0.11	0.66	0.91	0.71	0.14	0.52
831	*Quercus phellos*	NSL	0.12	0.79	0.64	0.66	0.08	0.50	0.13	0.64	0.79	0.70	0.14	0.50
832	*Quercus prinus*	NDH	0.48	0.92	0.71	0.95	0.39	0.80	0.47	0.72	0.92	0.95	0.37	0.79
833	*Quercus rubra*	WDH	0.30	0.77	0.64	0.92	0.32	0.64	0.29	0.62	0.74	0.86	0.40	0.62
834	*Quercus shumardii*	NSL	0.04	0.75	0.58	0.53	0.06	0.42	0.04	0.62	0.77	0.59	0.14	0.44
835	*Quercus stellata*	WDH	0.42	0.84	0.66	0.91	0.31	0.72	0.50	0.68	0.83	0.90	0.55	0.80
837	*Quercus velutina*	WDH	0.35	0.81	0.65	0.92	0.40	0.69	0.36	0.65	0.79	0.91	0.50	0.70
838	*Quercus virginiana*	NDH	0.35	0.90	0.62	0.89	0.52	0.71	0.44	0.66	0.91	0.87	0.40	0.74
842	*Quercus incana*	NSL	0.06	0.62	0.41	0.47	0.16	0.34	0.06	0.43	0.63	0.33	0.18	0.32
901	*Robinia pseudoacacia*	NDH	0.11	0.87	0.67	0.78	0.26	0.56	0.11	0.65	0.87	0.66	0.18	0.51
912	*Sabal palmetto*	NDH	0.27	0.96	0.61	0.84	0.57	0.68	0.31	0.63	0.95	0.84	0.38	0.67
921	*Salix amygdaloides*	NSLX	−0.01	0.57	0.34	0.19	0.11	0.25	−0.02	0.47	0.58	0.43	0.18	0.31
922	*Salix nigra*	NSH	0.01	0.77	0.60	0.01	0.08	0.39	0.03	0.59	0.8	0.47	0.16	0.42
931	*Sassafras albidum*	WSL	0.14	0.70	0.61	0.74	0.18	0.50	0.14	0.62	0.69	0.62	0.27	0.50
935	*Sorbus americana*	NSL	0.05	0.42	0.18	0.28	0.12	0.17	0.07	0.15	0.39	0.40	0.32	0.18
951	*Tilia americana*	WSL	0.19	0.83	0.63	0.78	0.27	0.58	0.22	0.62	0.83	0.88	0.48	0.62
971	*Ulmus alata*	WDL	0.25	0.85	0.71	0.86	0.25	0.65	0.29	0.72	0.85	0.89	0.13	0.65
972	*Ulmus americana*	WDH	0.14	0.73	0.57	0.88	0.38	0.53	0.22	0.59	0.72	0.91	0.36	0.56
973	*Ulmus crassifolia*	NDH	0.14	0.91	0.58	0.82	0.08	0.51	0.22	0.64	0.93	0.87	0.37	0.63
975	*Ulmus rubra*	WSL	0.08	0.76	0.60	0.47	0.41	0.51	0.10	0.61	0.76	0.60	0.30	0.49
977	*Ulmus thomasii*	NSLX	−0.01	0.65	0.31	0.40	0.10	0.25	−0.01	0.45	0.71	0.48	0.19	0.32

**Table A2:** Confident values among Importance Value classes for DISTRIB‐10 and DISTRIB‐hybrid models for 135 tree species. Confidence values are a combination of the percentage of RF trees predicting a mean value within ± 1 standard deviation of the mean or the median absolute deviation among the 1,001 regression trees

FIA Code	Scientific Name	Model Reliability	IV 0	IV 1–3	IV 4–6	IV 7–10	IV 11–20	IV 21–30	IV 31–50	IV 51–100	Model Reliability	IV 0	IV 1–3	IV 4–6	IV 7–10	IV 11–20	IV 21–30	IV 31–50	IV 51–100
12	*Abies balsamea*	High	0.841	0.971	0.821	0.762	0.734	0.725	0.698	0	High	0.819	0.969	0.818	0.765	0.74	0.726	0.695	0
43	*Chamaecyparis thyoides*	Low	0.659	0.984	0.662	0.542	0.468	0.53	0	0	Low	0.686	0.983	0.647	0.535	0.492	0.518	0	0
61	*Juniperus ashei*	High	0.661	0.992	0.908	0.866	0.803	0.721	0.657	0.745	High	0.809	0.991	0.908	0.862	0.788	0.718	0.68	0.748
68	*Juniperus virginiana*	Medium	0.909	0.964	0.838	0.726	0.626	0.583	0.577	0	Medium	0.881	0.956	0.844	0.742	0.641	0.597	0.619	0.521
71	*Larix laricina*	High	0.777	0.965	0.772	0.657	0.653	0.679	0.729	0.677	High	0.788	0.963	0.777	0.665	0.654	0.689	0.729	0.676
94	*Picea glauca*	Medium	0.687	0.962	0.752	0.619	0.504	0.437	0	0	Medium	0.861	0.958	0.762	0.615	0.53	0.477	0	0
95	*Picea mariana*	High	0.668	0.969	0.815	0.725	0.69	0.712	0.681	0.746	High	0.575	0.966	0.81	0.726	0.685	0.706	0.685	0.751
97	*Picea rubens*	High	0.862	0.981	0.827	0.766	0.726	0.672	0.63	0	High	0.824	0.981	0.832	0.774	0.725	0.67	0.657	0
105	*Pinus banksiana*	Medium	0.684	0.978	0.811	0.716	0.682	0.636	0.605	0	Medium	0.846	0.977	0.808	0.729	0.685	0.618	0.615	0
107	*Pinus clausa*	Medium	0.651	0.987	0.897	0.803	0.632	0.611	0.562	0.772	Medium	0.818	0.986	0.898	0.804	0.642	0.618	0.592	0.744
110	*Pinus echinata*	High	0.934	0.959	0.782	0.691	0.657	0.697	0.705	0.69	High	0.84	0.953	0.792	0.7	0.657	0.692	0.707	0.687
111	*Pinus elliottii*	High	0.733	0.981	0.89	0.854	0.787	0.702	0.701	0.711	High	0.747	0.979	0.88	0.849	0.781	0.706	0.713	0.725
115	*Pinus glabra*	Low	0.797	0.963	0.522	0.478	0.411	0	0	0	Low	0.67	0.961	0.527	0.499	0.584	0	0	0
121	*Pinus palustris*	Medium	0.791	0.975	0.872	0.755	0.625	0.589	0.664	0	Medium	0.874	0.973	0.871	0.765	0.635	0.594	0.66	0
123	*Pinus pungens*	Low	0.731	0.978	0.568	0.399	0.445	0	0	0	Low	0.73	0.976	0.542	0.459	0	0	0	0
125	*Pinus resinosa*	Medium	0.94	0.972	0.815	0.703	0.629	0.637	0.628	0	Medium	0.795	0.967	0.815	0.714	0.632	0.64	0.627	0
126	*Pinus rigida*	High	0.943	0.976	0.657	0.618	0.698	0.66	0.678	0.755	High	0.829	0.974	0.658	0.613	0.664	0.677	0.691	0.738
128	*Pinus serotina*	Medium	0.719	0.978	0.791	0.646	0.618	0.616	0.617	0.67	Medium	0.749	0.975	0.812	0.647	0.625	0.635	0.609	0.681
129	*Pinus strobus*	Medium	0.844	0.965	0.817	0.711	0.665	0.667	0.65	0	High	0.871	0.957	0.823	0.724	0.667	0.66	0.667	0
131	*Pinus taeda*	High	0.79	0.981	0.892	0.853	0.782	0.726	0.736	0.735	High	0.83	0.977	0.891	0.847	0.78	0.728	0.738	0.736
132	*Pinus virginiana*	High	0.89	0.974	0.819	0.709	0.645	0.604	0.507	0	High	0.952	0.971	0.823	0.713	0.648	0.593	0.511	0
221	*Taxodium distichum*	Medium	0.939	0.967	0.716	0.59	0.565	0.628	0.67	0	Medium	0.715	0.964	0.727	0.62	0.567	0.627	0.661	0
222	*Taxodium ascendens*	Medium	0.748	0.976	0.821	0.727	0.649	0.54	0.634	0.717	Medium	0.76	0.974	0.836	0.731	0.68	0.542	0.625	0.814
241	*Thuja occidentalis*	High	0.696	0.971	0.837	0.744	0.684	0.655	0.654	0.572	High	0.849	0.968	0.838	0.751	0.686	0.647	0.662	0.578
261	*Tsuga canadensis*	Medium	0.916	0.962	0.797	0.711	0.682	0.633	0.523	0	High	0.837	0.958	0.801	0.72	0.684	0.635	0.51	0
311	*Acer barbatum*	Low	0.772	0.96	0.598	0.575	0.326	0	0	0	Low	0.809	0.958	0.568	0.559	0.327	0	0	0
313	*Acer negundo*	Low	0.918	0.964	0.733	0.614	0.539	0.482	0.485	0	Low	0.91	0.961	0.775	0.681	0.589	0.539	0.466	0
314	*Acer nigrum*	Low	0.967	0.986	0.373	0.392	0.319	0	0	0	Low	0.968	0.982	0.431	0.414	0.38	0	0	0
315	*Acer pensylvanicum*	Medium	0.814	0.921	0.609	0.455	0	0	0	0	Medium	0.797	0.921	0.614	0.482	0	0	0	0
316	*Acer rubrum*	High	0.844	0.941	0.815	0.766	0.728	0.706	0.665	0.52	High	0.862	0.927	0.823	0.781	0.734	0.701	0.671	0.537
317	*Acer saccharinum*	Low	0.926	0.976	0.799	0.596	0.478	0.44	0.415	0	Low	0.947	0.975	0.83	0.621	0.521	0.464	0.481	0
318	*Acer saccharum*	High	0.845	0.962	0.844	0.763	0.697	0.681	0.668	0.478	High	0.841	0.955	0.852	0.777	0.712	0.676	0.667	0.606
319	*Acer spicatum*	Low	0.645	0.922	0.51	0	0	0	0	0	Low	0.703	0.923	0.506	0	0	0	0	0
331	*Aesculus glabra*	Low	0.88	0.974	0.43	0.419	0.371	0	0	0	Low	0.859	0.972	0.497	0.411	0.411	0	0	0
332	*Aesculus flava*	Low	0.87	0.969	0.537	0.493	0.429	0	0	0	Low	0.83	0.968	0.532	0.534	0.435	0	0	0
356	*Amelanchier spp*.	Low	0.892	0.931	0.507	0.414	0.335	0	0	0	Low	0.91	0.928	0.512	0.412	0.357	0	0	0
367	*Asimina triloba*	Low	0.84	0.964	0.374	0.752	0.394	0	0	0	Low	0.895	0.959	0.377	0.592	0	0	0	0
371	*Betula alleghaniensis*	High	0.785	0.943	0.736	0.713	0.711	0.665	0	0	High	0.879	0.94	0.741	0.712	0.706	0.674	0	0
372	*Betula lenta*	High	0.734	0.963	0.775	0.705	0.65	0.534	0	0	High	0.89	0.962	0.782	0.706	0.65	0.578	0	0
373	*Betula nigra*	Low	0.862	0.972	0.476	0.424	0.448	0.497	0	0	Low	0.927	0.969	0.48	0.406	0.44	0.692	0	0
375	*Betula papyrifera*	High	0.807	0.945	0.776	0.71	0.692	0.678	0.525	0	High	0.789	0.939	0.782	0.713	0.686	0.692	0.515	0
379	*Betula populifolia*	Low	0.685	0.967	0.638	0.508	0.433	0	0	0	Low	0.814	0.965	0.625	0.506	0.462	0	0	0
381	*Sideroxylon lanuginosum ssp. lanuginosum*	Low	0.838	0.969	0.529	0.455	0.452	0.576	0	0	Low	0.765	0.963	0.58	0.503	0.446	0.589	0	0
391	*Carpinus caroliniana*	Low	0.875	0.93	0.559	0.52	0.465	0	0	0	Low	0.868	0.923	0.557	0.502	0.461	0	0	0
401	*Carya aquatica*	Low	0.903	0.971	0.64	0.55	0.539	0.548	0	0	Medium	0.875	0.969	0.708	0.599	0.565	0.583	0	0
402	*Carya cordiformis*	Low	0.905	0.952	0.571	0.479	0.449	0.497	0	0	Low	0.903	0.947	0.627	0.5	0.479	0.44	0	0
403	*Carya glabra*	Medium	0.929	0.941	0.693	0.599	0.546	0.699	0	0	Medium	0.888	0.93	0.705	0.602	0.557	0	0	0
404	*Carya illinoinensis*	Low	0.907	0.975	0.715	0.534	0.443	0.412	0.454	0	Low	0.847	0.972	0.748	0.596	0.527	0.451	0.432	0
405	*Carya laciniosa*	Low	0.932	0.975	0.436	0.36	0.378	0	0	0	Low	0.886	0.973	0.485	0.387	0.402	0	0	0
407	*Carya ovata*	Medium	0.918	0.95	0.683	0.591	0.525	0.423	0	0	Medium	0.904	0.943	0.718	0.619	0.559	0.54	0	0
408	*Carya texana*	Medium	0.774	0.97	0.74	0.664	0.626	0.565	0.578	0	High	0.859	0.968	0.749	0.679	0.632	0.55	0.545	0
409	*Carya alba*	Low	0.861	0.929	0.67	0.586	0.512	0.509	0	0	Medium	0.852	0.919	0.676	0.585	0.501	0.547	0	0
421	*Castanea dentata*	Unacceptable	0.789	0.949	0.684	0.262	0	0	0	0	unacceptable	0.733	0.948	0	0.281	0	0	0	0
452	*Catalpa speciosa*	Unacceptable	0.774	0.991	0.446	0.284	0.483	0.19	0	0	Low								
461	*Celtis laevigata*	Low	0.826	0.961	0.753	0.643	0.595	0.556	0.542	0	Medium	0.782	0.96	0.774	0.702	0.637	0.656	0.584	0
462	*Celtis occidentalis*	Low	0.931	0.963	0.729	0.573	0.507	0.5	0	0	Medium	0.911	0.961	0.795	0.68	0.589	0.527	0.504	0
471	*Cercis canadensis*	Low	0.878	0.942	0.497	0.443	0.488	0	0	0	Low	0.911	0.934	0.511	0.438	0.541	0	0	0
491	*Cornus florida*	Medium	0.834	0.907	0.57	0.475	0.58	0.443	0	0	Medium	0.835	0.897	0.579	0.481	0.536	0.731	0	0
521	*Diospyros virginiana*	Low	0.825	0.944	0.583	0.5	0.528	0.447	0	0	Low	0.811	0.937	0.58	0.475	0.444	0.473	0	0
531	*Fagus grandifolia*	Medium	0.894	0.95	0.74	0.68	0.67	0.687	0.693	0	High	0.888	0.943	0.752	0.687	0.667	0.691	0.685	0
541	*Fraxinus americana*	Medium	0.883	0.941	0.749	0.666	0.638	0.538	0.466	0	Medium	0.86	0.931	0.765	0.693	0.647	0.53	0.514	0
543	*Fraxinus nigra*	Medium	0.925	0.96	0.782	0.703	0.631	0.494	0	0	Medium	0.792	0.955	0.795	0.708	0.637	0.517	0	0
544	*Fraxinus pennsylvanica*	Low	0.865	0.945	0.743	0.614	0.57	0.508	0.446	0	Low	0.825	0.936	0.781	0.671	0.596	0.55	0.507	0
546	*Fraxinus quadrangulata*	Low	0.878	0.981	0.466	0.444	0.41	0	0	0	Low	0.873	0.978	0.525	0.468	0.419	0	0	0
551	*Gleditsia aquatica*	Unacceptable	0.783	0.978	0.387	0.279	0.212	0	0	0	Low	0.918	0.977	0.408	0.337	0.155	0	0	0
552	*Gleditsia triacanthos*	Low	0.888	0.968	0.67	0.494	0.443	0.418	0	0	Low	0.944	0.964	0.75	0.617	0.527	0.516	0.366	0
555	*Gordonia lasianthus*	Medium	0.834	0.974	0.727	0.57	0.53	0.553	0	0	Medium	0.706	0.971	0.737	0.588	0.542	0.572	0	0
571	*Gymnocladus dioicus*	Unacceptable	0.764	0.985	0.331	0.715	0.189	0	0										
580	*Halesia spp*.	Low	0.707	0.952	0.527	0	0	0	0	0	Low	0.738	0.958	0.504	0	0	0	0	0
591	*Ilex opaca*	Medium	0.74	0.935	0.668	0.599	0.575	0	0	0	Medium	0.8	0.931	0.671	0.621	0.583	0	0	0
601	*Juglans cinerea*	Unacceptable	0.835	0.977	0.351	0.35	0.682	0	0	0	Low	0.947	0.975	0.392	0.395	0.301	0	0	0
602	*Juglans nigra*	Low	0.906	0.959	0.725	0.581	0.526	0.432	0.49	0	Low	0.907	0.956	0.775	0.66	0.576	0.478	0.462	0
611	*Liquidambar styraciflua*	High	0.873	0.962	0.838	0.781	0.721	0.631	0.5	0	High	0.868	0.958	0.843	0.792	0.731	0.633	0.51	0
621	*Liriodendron tulipifera*	High	0.925	0.96	0.795	0.735	0.702	0.638	0.528	0	High	0.921	0.953	0.804	0.747	0.706	0.643	0.54	0
641	*Maclura pomifera*	Low	0.911	0.977	0.823	0.603	0.482	0.46	0.434	0	Medium	0.912	0.975	0.818	0.696	0.553	0.558	0.57	0
651	*Magnolia acuminata*	Low	0.773	0.952	0.55	0.568	0	0	0	0	Low	0.905	0.952	0.532	0.574	0	0	0	0
652	*Magnolia grandiflora*	Low	0.68	0.952	0.517	0.432	0.401	0	0	0	Low	0.698	0.95	0.486	0.447	0.491	0	0	0
653	*Magnolia virginiana*	Medium	0.894	0.951	0.716	0.654	0.611	0.515	0	0	Medium	0.912	0.947	0.723	0.647	0.613	0.525	0	0
654	*Magnolia macrophylla*	Low	0.775	0.968	0.616	0.637	0	0	0	0	Low	0.796	0.967	0.557	0.619	0	0	0	0
655	*Magnolia fraseri*	Low	0.879	0.958	0.584	0.442	0.487	0	0	0	Low	0.822	0.956	0.572	0.446	0.464	0	0	0
682	*Morus rubra*	Low	0.922	0.955	0.498	0.416	0.392	0.457	0	0	Low	0.912	0.946	0.677	0.552	0.494	0.491	0	0
691	*Nyssa aquatica*	Medium	0.771	0.978	0.692	0.531	0.548	0.593	0.691	0	Medium	0.848	0.975	0.708	0.557	0.517	0.563	0.752	0
693	*Nyssa sylvatica*	Medium	0.851	0.917	0.705	0.612	0.518	0.496	0	0	Medium	0.852	0.908	0.707	0.609	0.522	0.502	0	0
694	*Nyssa biflora*	Medium	0.781	0.969	0.794	0.675	0.603	0.551	0.435	0	Medium	0.742	0.967	0.806	0.683	0.61	0.57	0.425	0
701	*Ostrya virginiana*	Low	0.884	0.929	0.603	0.548	0.492	0	0	0	Low	0.854	0.918	0.614	0.551	0.489	0	0	0
711	*Oxydendrum arboreum*	High	0.792	0.936	0.697	0.705	0.667	0	0	0	High	0.89	0.933	0.701	0.693	0.679	0	0	0
721	*Persea borbonia*	Low	0.725	0.944	0.623	0.538	0.6	0.369	0	0	Low	0.767	0.94	0.639	0.552	0.597	0	0	0
722	*Planera aquatica*	Low	0.711	0.979	0.419	0.407	0.342	0.358	0	0	Low	0.719	0.977	0.472	0.407	0.387	0	0	0
731	*Platanus occidentalis*	Low	0.865	0.959	0.58	0.477	0.478	0.39	0	0	Low	0.906	0.952	0.599	0.507	0.473	0.428	0	0
741	*Populus balsamifera*	Medium	0.65	0.965	0.763	0.73	0.651	0.577	0.633	0	Medium	0.695	0.962	0.762	0.716	0.653	0.65	0.665	0
742	*Populus deltoides*	Low	0.944	0.979	0.715	0.528	0.418	0.392	0.69	0	Low	0.956	0.974	0.773	0.563	0.449	0.434	0.393	0
743	*Populus grandidentata*	Medium	0.858	0.952	0.706	0.601	0.573	0.522	0	0	Medium	0.8	0.946	0.715	0.602	0.574	0.544	0	0
746	*Populus tremuloides*	High	0.9	0.961	0.826	0.762	0.723	0.705	0.704	0.746	High	0.792	0.955	0.836	0.773	0.727	0.709	0.702	0.769
761	*Prunus pensylvanica*	Low	0.845	0.955	0.554	0.518	0.491	0	0	0	Low	0.922	0.954	0.582	0.478	0.5	0	0	0
762	*Prunus serotina*	Medium	0.852	0.927	0.765	0.706	0.665	0.62	0.617	0.512	Medium	0.793	0.916	0.777	0.705	0.674	0.647	0.616	0.495
763	*Prunus virginiana*	Unacceptable	0.77	0.958	0.448	0.299	0.361	0	0										
766	*Prunus americana*	Unacceptable	0.94	0.979	0.439	0.281	0.236	0.248	0										
802	*Quercus alba*	Medium	0.902	0.946	0.798	0.716	0.676	0.689	0.676	0	Medium	0.889	0.936	0.809	0.732	0.682	0.683	0.673	0
804	*Quercus bicolor*	Low	0.906	0.977	0.468	0.414	0.422	0.352	0	0	Low	0.951	0.974	0.542	0.453	0.398	0.37	0	0
806	*Quercus coccinea*	Medium	0.917	0.952	0.713	0.652	0.615	0.531	0.456	0	Medium	0.888	0.947	0.721	0.652	0.62	0.601	0.465	0
809	*Quercus ellipsoidalis*	Medium	0.774	0.979	0.762	0.663	0.636	0.595	0	0	Medium	0.829	0.973	0.769	0.683	0.641	0.6	0	0
812	*Quercus falcata*	Medium	0.848	0.94	0.731	0.628	0.542	0.488	0	0	Medium	0.778	0.933	0.735	0.634	0.542	0.484	0	0
813	*Quercus pagoda*	Medium	0.727	0.954	0.63	0.584	0.562	0.374	0	0	Medium	0.874	0.949	0.644	0.6	0.555	0	0	0
816	*Quercus ilicifolia*	Unacceptable	0.962	0.98	0.298	0.961	0	0.704	0										
817	*Quercus imbricaria*	Low	0.84	0.978	0.69	0.569	0.496	0.408	0.475	0	Medium	0.954	0.977	0.73	0.657	0.552	0.418	0.5	0
819	*Quercus laevis*	Medium	0.702	0.976	0.729	0.598	0.593	0.607	0	0	Medium	0.83	0.973	0.75	0.623	0.585	0.604	0	0
820	*Quercus laurifolia*	Medium	0.842	0.955	0.791	0.696	0.616	0.573	0.559	0	Medium	0.75	0.952	0.8	0.704	0.619	0.6	0.588	0
822	*Quercus lyrata*	Low	0.877	0.968	0.657	0.555	0.536	0.494	0	0	Medium	0.89	0.965	0.693	0.582	0.553	0.601	0.357	0
823	*Quercus macrocarpa*	Medium	0.927	0.974	0.797	0.676	0.599	0.58	0.548	0	Medium	0.942	0.968	0.83	0.73	0.648	0.572	0.605	0
824	*Quercus marilandica*	Medium	0.821	0.964	0.759	0.68	0.625	0.538	0.513	0	Medium	0.804	0.958	0.766	0.669	0.604	0.62	0.566	0
825	*Quercus michauxii*	Low	0.798	0.955	0.446	0.414	0.438	0	0	0	Low	0.908	0.952	0.426	0.483	0.46	0	0	0
826	*Quercus muehlenbergii*	Low	0.939	0.966	0.624	0.596	0.49	0	0	0	Medium	0.876	0.959	0.664	0.594	0.535	0	0	0
827	*Quercus nigra*	High	0.693	0.95	0.828	0.734	0.662	0.512	0.434	0	High	0.883	0.943	0.836	0.745	0.662	0.528	0.4	0
828	*Quercus texana*	Low	0.736	0.977	0.654	0.526	0.599	0.412	0	0	Low	0.791	0.977	0.724	0.595	0.636	0.441	0.364	0
830	*Quercus palustris*	Low	0.878	0.981	0.682	0.505	0.446	0.455	0	0	Low	0.891	0.98	0.716	0.561	0.501	0.444	0	0
831	*Quercus phellos*	Low	0.761	0.954	0.652	0.556	0.517	0.469	0	0	Low	0.829	0.949	0.676	0.559	0.547	0.464	0	0
832	*Quercus prinus*	High	0.84	0.971	0.809	0.735	0.699	0.686	0.639	0	High	0.918	0.97	0.817	0.742	0.703	0.672	0.662	0
833	*Quercus rubra*	Medium	0.898	0.936	0.759	0.678	0.664	0.555	0.564	0	Medium	0.88	0.926	0.778	0.695	0.659	0.553	0.61	0
834	*Quercus shumardii*	Low	0.811	0.971	0.483	0.425	0.421	0	0	0	Low	0.818	0.966	0.506	0.454	0.419	0	0	0
835	*Quercus stellata*	High	0.822	0.951	0.817	0.764	0.705	0.671	0.664	0.701	High	0.771	0.941	0.812	0.76	0.708	0.686	0.702	0.705
837	*Quercus velutina*	Medium	0.892	0.946	0.744	0.668	0.668	0.666	0.637	0	High	0.875	0.936	0.763	0.687	0.668	0.675	0.649	0
838	*Quercus virginiana*	High	0.664	0.974	0.88	0.817	0.732	0.628	0.634	0.699	High	0.627	0.971	0.875	0.822	0.742	0.677	0.665	0.686
842	*Quercus incana*	Low	0.827	0.97	0.499	0.472	0.384	0	0	0	Low	0.713	0.967	0.533	0.499	0.426	0	0	0
901	*Robinia pseudoacacia*	Medium	0.942	0.971	0.757	0.616	0.51	0.418	0.325	0	Low	0.929	0.964	0.775	0.627	0.52	0.456	0.34	0
912	*Sabal palmetto*	Medium	0.711	0.983	0.896	0.857	0.707	0.617	0.582	0.67	Medium	0.583	0.981	0.881	0.849	0.75	0.665	0.612	0.546
921	*Salix amygdaloides*	Unacceptable	0.825	0.986	0.381	0.323	0.266	0.235	0										
922	*Salix nigra*	Low	0.938	0.973	0.652	0.483	0.439	0.395	0.357	0	Low	0.926	0.966	0.707	0.517	0.494	0.456	0.436	0
931	*Sassafras albidum*	Low	0.859	0.942	0.667	0.559	0.473	0.536	0	0	Low	0.869	0.934	0.679	0.586	0.498	0.563	0	0
935	*Sorbus americana*	Low	0.711	0.93	0	0.308	0	0	0	0	Low	0.707	0.931	0.382	0	0	0	0	0
951	*Tilia americana*	Medium	0.929	0.953	0.706	0.63	0.587	0.492	0.562	0	Medium	0.906	0.944	0.728	0.636	0.612	0.548	0	0
971	*Ulmus alata*	Medium	0.855	0.942	0.757	0.692	0.586	0.506	0	0	Medium	0.775	0.934	0.762	0.694	0.612	0.516	0.381	0
972	*Ulmus americana*	Low	0.846	0.933	0.751	0.637	0.605	0.525	0.51	0	Medium	0.807	0.925	0.786	0.705	0.642	0.555	0.503	0
973	*Ulmus crassifolia*	Low	0.712	0.973	0.862	0.721	0.567	0.509	0.516	0	Medium	0.786	0.973	0.856	0.77	0.653	0.583	0.503	0
975	*Ulmus rubra*	Low	0.908	0.944	0.581	0.536	0.465	0.4	0	0	Low	0.889	0.936	0.632	0.538	0.495	0.448	0	0
977	*Ulmus thomasii*	Unacceptable	0.855	0.982	0.277	0.261	0	0	0										
	Average	All	0.829	0.961	0.666	0.585	0.515	0.404	0.258	0.090	All	0.837	0.957	0.682	0.597	0.523	0.401	0.273	0.094
		High	0.805	0.964	0.811	0.747	0.706	0.637	0.556	0.366	High	0.832	0.959	0.808	0.742	0.699	0.642	0.571	0.308
		Medium	0.831	0.957	0.753	0.657	0.594	0.537	0.364	0.078	Medium	0.828	0.953	0.758	0.663	0.591	0.517	0.340	0.081
		Low	0.838	0.961	0.586	0.496	0.424	0.254	0.100	0.000	Low	0.850	0.960	0.569	0.476	0.388	0.193	0.073	0.000

**Table A3:** Overall predictor variable importance for 45 environmental data layers used to model suitable habitat of 135 tree species among DISTRIB‐hybrid grids. Values have been normalized to a 0–100 scale

	SumVarImp^1^	SumRankRecip^2^	FreqTop10^3^	VarImpIndex^4^
*Climate*				
PANN	84.2	54.7	80.2	73
PGrow	74.6	39.4	52.3	55.4
TANN	96.3	66	98.8	87
TGrow	95.1	62.5	94.2	83.9
TSUMavg	99.4	100	100	99.8
TWINavg	100	92.3	98.8	97.1
Aridity	76.6	42.6	61.6	60.3
*Elevation*				
ElvCV	58	11.5	12.8	27.4
ElvMAX	67.9	23.6	29.1	40.2
ElvMEAN	70.9	30	34.9	45.3
ElvMEDIAN	61	15.9	15.1	30.7
ElvMIN	61.5	15.1	14	30.2
ElvRANGE	64.5	19.9	25.6	36.7
ElvStdDev	73.3	34.1	38.4	48.6
*Geographic*				
DayLenCV	90.7	51.7	77.9	73.5
*Soil properties*				
AWC	68.3	30.5	34.9	44.6
AWS	65.5	23.7	23.3	37.5
BD3RDBAR	65.6	26.3	29.1	40.3
CACO3	44.7	11.6	10.5	22.2
CEC7	63.5	20.1	25.6	36.4
DEP2WATTBL	70.1	28.5	32.6	43.7
KFACTRF	65.5	20.5	22.1	36
TFACTOR	62.7	19.9	23.3	35.3
KSAT	69.5	41.1	43	51.2
OM	69.6	27.5	34.9	44
CLAYEY	30.6	15	12.8	19.5
LOAMY	53.6	10.2	11.6	25.2
SANDY	50.9	6.4	4.7	20.6
OTHER	44	11.3	8.1	21.1
CLAY	65.2	25.3	24.4	38.3
SAND	65.9	28.7	30.2	41.6
SILT	68.1	34.9	37.2	46.8
PH	84.1	51.1	66.3	67.2
SIEVE10	74.1	43.9	44.2	54.1
SIEVE200	67	24.2	27.9	39.7
SProd	72.7	46.1	43	54
*Soil type*				
Alfisols	57.3	34.3	29.1	40.2
Aridisols	0	0	0	0
Entisols	17.1	5	3.5	8.5
Histosols	29.5	5.8	8.1	14.5
Inceptisol	62.6	29	27.9	39.8
Mollisols	44.7	24.8	27.9	32.5
Spodosols	24	8.7	11.6	14.8
Ultisols	46.6	25.4	30.2	34.1
Vertisols	18.5	13.2	8.1	13.3

Note

Variable names are described in Table 1. SumVarImp1 = sum of predictor importance scores from Random Forest (percent increase) across all species. SumRankRecip2 = sum of the reciprocal of rank of each predictor across all species. FreqTop103 = frequency of the top 10 predictors with the highest importance across all species. VarImpIndx4 = Variable Importance Index is overall mean importance defined by three normalized metrics which indicates the variables influence in models for the 135 species.

**Table A4:** The number of FIA inventory plots corresponding to the hybrid lattice and uniform grid cells for the region depicted in Figure 4 E and F

FIA Plots	DISTRIB‐hybrid		DISTRIB−10	
	Number of cells	Area (km^2^)	Number of cells	Area (km^2^)
0	109	27,600	529	52,900
1	162	30,900	316	31,600
2–3	461	59,000	482	48,200
4–6	792	91,300	768	76,800
7–9	517	51,800	509	50,900
10–12	51	5,000	50	5,000
13	1	100	1	100
Total	2,093	265,700	2,655	265,500
